# Teaching strategies for promoting female college students' physical activity

**DOI:** 10.3389/fpsyg.2025.1569578

**Published:** 2025-07-02

**Authors:** Ji Ma, Yuqiang Guo, Chao Zhu, Chunyuan Wen, Qiaoqiao Deng, Xiao Ma

**Affiliations:** School of Physical Education, Shanghai University of Sport, Shanghai, China

**Keywords:** gender roles, exercise involvement, sports projects, physical activity, female undergraduates

## Abstract

**Background:**

Although physical activity (PA) levels among female students have improved in primary and secondary education, female undergraduates consistently show lower PA levels compared to males and younger female students. Previous studies suggest that gender stereotypes and socially constructed traits significantly impact females' choices and engagement in sports. This study examines how gender roles and sports type choices influence PA levels and physical exercise involvement among female undergraduates.

**Method:**

Cluster sampling was used to recruit 500 undergraduates (256 males, 244 females) from five universities in Shanghai. Valid data from 439 undergraduates (mean age = 19.02 ± 1.04 years) were collected using the Physical Activity Rating Scale (PARS-3), Chinese College Students' Sex-Role Inventory (CSRI-50), and Physical Exercise Involvement Scale (PEIS). Data were analyzed through descriptive statistics, chi-square tests, and ANOVA.

**Results:**

Female undergraduates had significantly lower PA and physical exercise involvement scores compared to male peers (PA: *p* < 0.001, Cohen's *d* = 0.494; involvement: *p* < 0.001, Cohen's *d* = 0.752). No significant gender differences were observed in sports choices (*p* = 0.402). Female undergraduates showed the lowest PA levels in masculine sports (*p* = 0.002). Among female participants, gender roles significantly affected PA and involvement, with androgynous individuals performing best, especially in masculine sports (PA: *p* < 0.001; involvement: *p* < 0.001). Feminine and undifferentiated roles were associated with lower scores in value cognition (*p* < 0.001) and autonomy (*p* < 0.001).

**Conclusion:**

Gender stereotypes significantly impact female undergraduates' PA levels and physical exercise involvement. Promoting androgynous traits and reducing gender-role constraints in educational contexts can enhance female undergraduates' PA engagement. Future educational strategies should emphasize gender inclusivity and autonomy to address existing gender-related disparities in sports participation.

## 1 Introduction

It is well established that female students are involved in a lower level of physical activity (PA) compared to their male peers, which leads to unsatisfactory physical fitness and a higher health risk of being affected by various non-communicable diseases or experiencing discomfort and pain (Melzer et al., 2010; Oguma and Shinoda-Tagawa, 2004; Watson-Mackie et al., 2024). Benefiting from a series of PA intervention programs and curriculum reforms, the physical activity levels of female students in primary and secondary education have shown notable improvement over the past three decades (Landry and Driscoll, 2012; Neil-Sztramko et al., 2021). However, limited efforts have been made to address the physical activity levels of female undergraduates, and related reports remain scarce. It is believed that undergraduates gained a higher PA level compared to their early ages since they already well understand how regular exercise benefits their lives and fosters PA-related habits. Moreover, they can schedule their study (Cullen and Oppenheimer, 2024), physical exercises, and entertainment more flexibly than their early ages, so they spend more time on physical exercise and have a higher level of daily PA (Favieri et al., 2023). Therefore, it is unnecessary to carry out a specific intervention program for PA promotion to avoid impairing their self-determined physical exercise behaviors (Pan et al., 2022). However, existing evidence shows a controversial fact that female undergraduates maintain lower levels of PA compared with students in earlier stages, including primary and secondary education (Luo et al., 2010; Deforche et al., 2015), and also lower than their male peers (Ricardo et al., 2022). Daily PA analysis suggested that female postgraduates stay at lower PA levels than their male peers even if they spend equal time on living-related PA and PE courses, which indicated the low level of PA depends not only on PA-related time expenditure but also on their PA involvement and other relavent factors.

Generally, gender differences in PA remain steady and are attributed to gender-related physiological and genetic differences (Hunter et al., 2023). It also suggested that female undergraduates spend less time on physical exercise because they perceived higher pressures of academic pursuit and job finding (Yang et al., 2017) which led to the unsatisfactory PA-related habits fostered. However, more recent research suggests that stereotypes may be the primary factor influencing young women's participation in sports (Archer and McDonald, 1990; Liu et al., 2023) via project choices and exercise habits. These stereotypes are rooted in socially constructed gender roles and expectations (Wei et al., 2024), announced as “masculine” or “feminine” traits, respectively for males and females (Liu et al., 2011). Traditionally, men are generally expected to exhibit traits such as bravery, competitiveness, and strength, while women are expected to show gentleness, empathy, and cleanliness (Bloss, 2017; Naples et al., 2016). As social progress, females increasingly engage in professional careers and shoulder more social responsibility. As a consequence, female's gender roles have significantly changed in line with socially expected traits. Meanwhile, the influence of socially expected traits is amplified by the diversification of social media and communication channels (Wood and Fixmer-Oraiz, 2018). The most significant change in female socially expected traits is the involvement of male's socially expected traits. Alternatively, it could be understood as women are exhibiting traits traditionally associated with men, while men are showing greater social adaptability by adopting some traditionally female traits (Sumra, 2019). Consequently, gender role theory introduced the concept of “androgyny” to describe individuals who possess a blend of both male and female advantageous traits, reflecting the compatibility of non-typical gender roles (Ashmore, 1998; John et al., 2010). Subsequent evidence has supported that androgynous individuals demonstrate more advantages in social life and competition (Dong, 2009).

Similar changes occur in sports. Traditional, competitive sports require individuals to display traits that align with socially approved masculine gender roles, while aesthetically oriented sports require individuals to embody traits associated with feminine gender roles (Yang et al., 2017). So the sports can be categorized as masculine sports, Typical “masculine sports” include basketball, football, ice hockey, and kickboxing, while typical “feminine sports” include artistic gymnastics, aerobics, dance sports, and yoga (gender-typing sports: social representations of masculine, feminine, and gender-neutral sports among US university students) (Hardin and Greer, 2009). Early results show that males are more willing to engage in competitive sports to highlight traits such as competitiveness, proactivity, and initiative, while females tend to participate more in aesthetically oriented sports to display grace and elegance. This stereotype often causes women in the “feminine” group to experience anxiety and discomfort when participating in “masculine sports,” affecting their enthusiasm and satisfaction with PA (Guillet et al., 2006). However, recent studies show that the disparity in preferences for sports is narrowing, with an increasing number of neutral sports being jointly participated in by both males and females, including martial arts, swimming, volleyball, badminton, table tennis, and tennis (Clément-Guillotin and Fontaine, 2010; Koivula, 2001). This may be related to the emergence of androgyny, as women exhibiting androgynous traits tend to choose a broader range of sports, exhibit more disciplined exercise behaviors, and have more harmonious sports-related social interactions (Dong, 2017). By contrast, groups combining both negative male and female traits are often associated with negative exercise tendencies, with these individuals referred to as “undifferentiated” (Dong and Zhang, 2016).

According to the shortage of female PA and the potential cause of socially expected traits, the present study surveyed undergraduates in grade one and grade two, who still have compulsory PE course and optional PE courses, to describe the differences between male and female undergraduates on sports project choices, extracurricular PA, and in-class exercise involvement, and then analyze whether the varies of socially expected traits are associated with females undergraduate's PA level. It would contribute to proposing strategies to enhance the PA levels of female undergraduates.

## 2 Method

### 2.1 Participants

There were 500 undergraduates who participated in the present study, recruited through cluster sampling from five universities in Shanghai, including two comprehensive universities, one university of technology, one normal university, and one university of finance and economics. Among the participants, 256 were male and 244 were female. After a rigorous data cleaning process, cases with missing responses or patterned answers were excluded, leaving 439 valid cases for analysis. The effective response rate was 87.8%. The average age of the analyzed sample was 19.02 ± 1.04 years, and the academic disciplines included literature (21.9%), science (31.2%), engineering (35.5%), and education (11.4%).

### 2.2 Measurement tools

This study used the Physical Activity Rating Scale (PARS-3) developed by Liang (1994) to investigate undergraduates' extracurricular PA. The PARS-3 was selected due to its widespread use in assessing physical activity levels among Chinese undergraduates and its strong psychometric properties. It offers a simple but effective composite score reflecting overall exercise behavior, which aligns with the objectives of this study to quantify extracurricular physical activity. The scale was used to assess participants' exercise volume based on three dimensions: intensity, duration, and frequency. Following the research objectives, the exercise behavior of participants was evaluated using their exercise volume scores, calculated with the formula “Intensity × Duration × Frequency” (with a maximum score of 100 and a minimum score of 0). Each dimension was divided into five levels: Intensity and frequency were rated from 1 to 5 points, while duration was rated from 0 to 4 points. The scale demonstrated a Cronbach's α of 0.839, split-half reliability of 0.816, and item-total correlations ranging from 0.658 to 0.842 (*p* < 0.01).

This study used the Chinese College Students' Sex-Role Inventory: 50 Items (CSRI-50) (Liu et al., 2011) to measure the gender role characteristics of undergraduates. The CSRI-50 was selected due to its strong theoretical foundation based on Bem's gender schema theory and its demonstrated suitability for Chinese college populations. It has been widely applied in gender role studies across various disciplines in China, providing a culturally relevant and psychometrically sound measure. The scale consisted of 50 items and used a 7-point Likert scale (1–7 points ranging from “strongly disagree” to “strongly agree”). The CSRI-50 included three subscales: masculine (16 items), feminine (16 items), and androgynous (18 items). Gender role types were categorized based on the median scores of the masculine (*M*) and feminine (*F*) subscales: undifferentiated (*M* < 4.8, *F* < 5.0), feminine (*M* < 4.8, *F* ≥ 5.0), masculine (*M* ≥ 4.8, *F* < 5.0), and androgynous (*M* ≥ 4.8, *F* ≥ 5.0). Cronbach's α for the total scale was set at 0.953, and the split-half reliability was set at 0.877. Cronbach's α values for the masculine and feminine subscales were 0.889 and 0.845, respectively, with KMO values of 0.929 and 0.932, indicating good reliability and validity of the scale.

The Physical Exercise Involvement Scale for College Students (PEIS) (Dong, 2017) was used to measure undergraduates' involvement in physical exercise. This scale was chosen for its comprehensive structure and proven applicability among Chinese undergraduates. It captures not only behavioral engagement but also the cognitive and emotional dimensions of exercise participation, aligning closely with the study's aim to explore undergraduates' deeper involvement in physical activity beyond frequency and duration. The scale consisted of 20 items and was divided into four dimensions: vitality persistence (6 items), focused satisfaction (6 items), value recognition (5 items), and participation autonomy (3 items). It used a 5-point Likert scale (1–5 points ranging from “strongly disagree” to “strongly agree”), with higher scores indicating greater physical exercise involvement. Cronbach's α for the total scale was set at 0.906, the split-half reliability at 0.898, the test-retest reliability at 0.892, and the KMO value at 0.916. The Cronbach's α values for the subscales ranged from 0.899 to 0.945, and the split-half reliability ranged from 0.883 to 0.926, indicating good reliability and validity of the scale.

### 2.3 Statistical analysis

All statistical analyses were conducted using SPSS software (version 26.0; SPSS Inc., Chicago, IL, USA). Measures of central tendency were calculated to provide an overview of the data. Additionally, difference tests were performed to examine interactions among gender role and project type, PA, and physical exercise involvement. Specifically, *t*-tests and ANOVA were employed for enumeration data, whereas χ^2^ tests were utilized for proportional data. To minimize potential common method bias, Harman's single-factor test was applied in this study. The results indicated that the first factor accounted for 31.05% of the variance, which is below the threshold of 40%, thus confirming the absence of significant common method bias.

## 3 Results

### 3.1 Descriptive statistics

Examination of skewness and kurtosis revealed that all variables had absolute values within acceptable ranges. The maximum skewness was 0.454 (well below the threshold of 2), and the maximum kurtosis was 0.712 (below the threshold of 7). These findings suggest that the distributions of all variables were approximately normal, thereby meeting the assumption of normality required for parametric analyses.

Gender differences in PA and physical exercise involvement, along with its four dimensions, were analyzed using independent samples *t*-tests. The results showed that male undergraduates gained a significant higher PA score according to the PARS-3 than that of female undergraduates (*t* = 5.170*, p* < 0.001, Cohen's *d* = 0.494; *M*_*male*_ ± *SD*_*male*_ = 19.29 ± 9.466, *M*_*female*_ ± *SD*_*female*_ = 16.89 ± 9.138). Similar differences could also be seen on the score of physical involvement gain from PEIS (*t* = 7.877, *p* < 0.001, Cohen's *d* = 0.752; *M*_*male*_ ± *SD*_*male*_ = 57.55 ± 17.283, *M*_*female*_ ± *SD*_*female*_ = 44.05 ± 18.523). Sub-scale scores indicated male undergraduates showed higher persistence (*t* = 6.470, *p* < 0.001, Cohen's *d* = 0.618; *M*_*male*_ ± *SD*_*male*_ = 17.58 ± 6.337, *M*_*female*_ ± *SD*_*female*_ = 13.54 ± 6.734), concentration (*t* = 6.019, *p* < 0.001, Cohen's *d* = 0.575; *M*_*male*_ ± *SD*_*male*_ = 16.64 ± 6.987, *M*_*female*_ ± *SD*_*female*_ = 12.77 ± 6.493), value cognition (*t* = 5.846, *p* < 0.001, Cohen's *d* = 0.558; *M*_*male*_ ± *SD*_*male*_ = 14.05 ± 52,846, *M*_*female*_ ± *SD*_*female*_ = 11.77 ± 6.442), and participation autonomy (*t* = 5.105, *p* < 0.001, Cohen's *d* = 0.488; *M*_*male*_ ± *SD*_*male*_ = 9.27 ± 3.723, *M*_*female*_ ± *SD*_*female*_ = 7.29 ± 4.372) on sports than that of female undergraduates (see Table 1).

**Table 1:** Gender differences in PA and exercise.

**Gender**	**PA**				**Involvement**			
	***M*** ±***SD***	***t***	***p***	**Cohen's** ***d***	***M*** ±***SD***	***t***	***p***	**Cohen's** ***d***
Male	19.29 ± 9.466	5.170	0.000	0.494	57.55 ± 17.283	7.877	0.000	0.752
Female	14.92 ± 8.247				44.05 ± 18.523			
**Gender**	**Persistence**				**Concentration**			
	***M*** ±***SD***	***t***	***p***	**Cohen's** ***d***	***M*** ±***SD***	***t***	***p***	**Cohen's** ***d***
Male	17.58 ± 6.337	6.470	0.000	0.618	16.64 ± 6.987	6.019	0.000	0.575
Female	13.54 ± 6.734				12.77 ± 6.493			
**Gender**	**Value cognition**				**Participation autonomy**			
	***M*** ±***SD***	***t***	***p***	**Cohen's** ***d***	***M*** ±***SD***	***t***	***p***	**Cohen's** ***d***
Male	14.05 ± 5.818	5.846	0.000	0.558	9.27 ± 3.723	5.105	0.000	0.488
Female	10.65 ± 6.324				7.29 ± 4.372			

According to sports choice, 41.2% of male undergraduates (*n* = 87) chose gender-neutral sports, then masculine (*n* = 70, 33.2%) and feminine ones (*n* = 54, 25.6%), while the number of female undergraduates on each option were about the same, showing as gender-neutral (*n* = 81, 35.5%), masculine (*n* = 78, 34.2%) and feminine sports (*n* = 69, 30.3%). There was no significant gender-related difference in the sports choices of undergraduates [$\chi_{\left(2,438\right)}^{2}$ = 1.820, *p* = 0.402, Cramér's *V* = 0.064] (see Tables 2, 3).

**Table 2:** Results of the chi-square test.

**Variable interactions**		**d*f***	**χ^2^**	**Cramér's *V***	***p***
Gender ^*^ Project type		2	1.820	0.064	0.402
Gender ^*^ Gender Role		3	95.601	0.269	0.000
Gender Role^*^ Project type	Male	6	9.457	0.086	0.149
	Female		8.190	0.077	0.225

**Table 3:** Distribution of gender, gender roles, and project types.

**Gender**	**Gender role**	**Project type**			**Total**
		**Masculine sports**	**Feminine sports**	**Gender-neutral sports**	
Male	Androgynous	24 (31.6%)	19 (25.0%)	33 (43.4%)	76 (36.0%)
	Feminine	12 (57.1%)	3 (14.3%)	6 (28.6%)	21 (10.0%)
	Masculine	20 (25.0%)	25 (31.3%)	35 (43.8%)	80 (37.9%)
	Undifferentiated	14 (41.2%)	7 (20.6%)	13 (38.2%)	34 (16.1%)
	Total	70 (33.2%)	54 (25.6%)	87 (41.2%)	211
Female	Androgynous	29 (40.3%)	20 (27.8%)	23 (31.9%)	72 (31.6%)
	Feminine	20 (23.3%)	31 (36.0%)	35 (40.7%)	86 (37.7%)
	Masculine	5 (41.7%)	2 (16.7%)	5 (41.7%)	12 (5.3%)
	Undifferentiated	24 (41.4%)	16 (27.6%)	18 (31.0%)	58 (25.4%)
	Total	78 (34.2%)	69 (30.3%)	81 (35.5%)	228

According to gender roles, the distribution among male undergraduates was as follows: masculine (*n* = 80, 37.9%), androgynous (*n* = 76, 36.0%), undifferentiated (*n* = 34, 16.1%), and feminine (*n* = 21, 10.0%). Among female undergraduates, the gender role distribution was as follows: feminine (*n* = 86, 37.7%), androgynous (*n* = 72, 31.6%), undifferentiated (*n* = 58, 25.4%), and masculine (*n* = 12, 5.3%). A significant gender-related difference was observed among the gender role of undergraduates [$\chi_{\left(3,438\right)}^{2}$ = 95.601, *p* < 0.001, Cramér's *V* = 0.269] (see Tables 2, 3).

A chi-square test was conducted to examine the differences in project selection based on gender roles among male undergraduates. The results showed that χ^2^_(6, 210)_ = 9.457, *p* = 0.149, Cramér's *V* = 0.086, indicating no significant differences in project selection across different gender roles. The project choices for the androgynous group were as follows: masculine sports: 24 (31.6%), feminine sports: 19 (25.0%), gender-neutral sports: 33 (43.4%); those for the feminine group were: masculine sports: 12 (57.1%), feminine sports: 3 (14.3%), and gender-neutral sports: 6 (28.6%); those for the masculine group were: masculine sports: 20 (25.0%), feminine sports: 25 (31.3%), and gender-neutral sports: 35 (43.8%); and those for the undifferentiated group were: masculine sports: 14 (41.2%), feminine sports: 7 (20.6%), and gender-neutral sports: 13 (38.2%) (see Tables 2, 3).

A chi-square test was also conducted to examine the differences in project selection based on gender roles among female undergraduates. No significant differences in project selection were observed across different gender roles [χ^2^_(6, 227)_ = 8.190, *p* = 0.225, Cramér's *V* = 0.077]. The project choices for the androgynous group were as follows: masculine sports: 29 (40.3%), feminine sports: 20 (27.8%), and gender-neutral sports: 23 (31.9%); those for the feminine group were: masculine sports: 20 (23.3%), feminine sports: 31 (36.0%), and gender-neutral sports: 35 (40.7%); those for the masculine group were: masculine sports: 5 (41.7%), feminine sports: 2 (16.7%), and gender-neutral sports: 5 (41.7%); and those for the undifferentiated group were: masculine sports: 24 (41.4%), feminine sports: 16 (27.6%), and gender-neutral sports: 18 (31.0%) (see Tables 2, 3).

### 3.2 Internal physical activity differences among female undergraduates

Levene's tests were conducted to examine the homogeneity of variances for all variables subjected to one-way ANOVA. None of the tests reached statistical significance (*p* > 0.05), suggesting that the assumption of equal variances was satisfied across all group comparisons.

A one-way analysis of variance (ANOVA) was conducted with project type as the independent variable and PA level as the dependent variable. The results showed *F*_(2, 227)_ = 6.682, *p* = 0.002, η^2^ = 0.056, indicating a statistically significant difference in PA levels between different projects, with a moderate to small effect size. *Post-hoc* multiple comparisons revealed that feminine sports (*M* = 17.32, *SD* = 8.608) had significantly higher PA levels than masculine sports (*M* = 12.49, *SD* = 7.742, *p* < 0.001). Gender-neutral sports (*M* = 15.21, *SD* = 7.840) were significantly higher than masculine sports (*p* = 0.034). No significant difference was found between feminine sports and gender-neutral sports (*p* = 0.111) (see Tables 4, 5).

**Table 4:** Comparison of PA across sports.

**Project type**	***M* ±*SD***	***df***	***F***	***p***	**η^2^**
Masculine sports	34.24 ± 15.654	2	20.530	0.000	0.154
Feminine sports	51.35 ± 19.096				
Gender-neutral sports	47.28 ± 16.658				

**Table 5:** *Post-hoc* multiple comparisons of PA across sports.

**Item**	**Mean difference**	***SE***	***p***	**95%** ***CI***	
				**Lower**	**Upper**
Masculine sports–Feminine sports	−17.102	2.828	0.000	−22.67	−11.53
Masculine sports–Gender-neutral sports	−13.038	2.714	0.000	−18.39	−7.69
Feminine sports–Gender-neutral sports	4.063	2.803	0.149	−1.46	9.59

Additionally, a one-way ANOVA was conducted on the PA levels of different gender roles in masculine sports. The results showed: *F*_(3, 227)_ = 7.272, *p* < 0.001, η^2^ = 0.228, indicating a statistically significant difference in PA levels between different gender roles, with a large effect size. *Post-hoc* multiple comparisons revealed that androgynous individuals (*M* = 16.72, *SD* = 8.730) had significantly higher PA levels than feminine (*M* = 9.70, *SD* = 5.723, *p* = 0.001) and undifferentiated (*M* = 8.92, *SD* = 5.672, *p* < 0.001) individuals. No significant difference was observed between feminine and masculine (*M* = 16.20, *SD* = 4.147, *p* = 0.065) or undifferentiated (*p* = 0.710) individuals, though masculine was significantly higher than undifferentiated (*p* = 0.036) (see Tables 6, 7).

**Table 6:** PA differences in masculine sports by gender role.

**Gender role**	***M* ±*SD***	***df***	***F***	***p***	**η^2^**
Androgynous	45.17 ± 15.699	3	16.796	0.000	0.405
Feminine	29.55 ± 12.137				
Masculine	44.80 ± 3.114				
Undifferentiated	22.83 ± 8.095				

**Table 7:** *Post-hoc* multiple comparisons of PA across gender roles.

**Item**	**Mean difference**	***SE***	***p***	**95%** ***CI***	
				**Lower**	**Upper**
Androgynous—Feminine	15.67	3.580	0.000	8.54	22.80
Androgynous—Masculine	0.31	5.964	0.959	−11.57	12.19
Androgynous—Undifferentiated	22.41	3.399	0.000	15.64	29.19
Feminine—Masculine	−15.36	6.158	0.015	−27.63	−3.09
Feminine—Undifferentiated	6.74	3.729	0.075	−0.69	14.17
Masculine—Undifferentiated	22.11	6.055	0.000	10.04	34.17

### 3.3 Internal physical exercise involvement differences among female undergraduate

Homogeneity of variances was assessed using Levene's tests prior to each ANOVA. All tests yielded non-significant results (*p* > 0.05), supporting the use of parametric procedures.

A one-way ANOVA was conducted with project type as the independent variable and physical exercise involvement as the dependent variable. The results indicated significant differences in physical exercise involvement across different project types: *F*_(2, 227)_ = 20.530, *p* < 0.001, η^2^ = 0.154, reflecting a strong effect size. *Post-hoc* multiple comparisons revealed that individuals participating in feminine sports (*M* = 51.35, *SD* = 19.096) demonstrated significantly higher physical exercise involvement than those participating in masculine sports (*M* = 34.24, *SD* = 15.654, *p* < 0.001). Similarly, individuals in gender-neutral sports (*M* = 47.28, *SD* = 16.658) reported significantly higher physical exercise involvement than those in masculine sports (*p* < 0.001). However, no significant difference was observed between participants in feminine sports and gender-neutral sports (*p* = 0.149) (see Tables 8, 9).

**Table 8:** Comparison of total exercise involvement score across sports.

**Project type**	***M* ±*SD***	**d*f***	***F***	***p***	**η^2^**
Masculine sports	34.24 ± 15.654	2	20.530	0.000	0.154
Feminine sports	51.35 ± 19.096				
Gender-neutral sports	47.28 ± 16.658				

**Table 9:** *Post-hoc* multiple comparisons of total exercise involvement score across sports.

**Item**	**Mean difference**	***SE***	***p***	**95%** ***CI***	
				**Lower**	**Upper**
Masculine sports–Feminine sports	−17.102	2.828	0.000	−22.67	−11.53
Masculine sports–Gender-neutral sports	−13.038	2.714	0.000	−18.39	−7.69
Feminine sports–Gender-neutral sports	4.063	2.803	0.149	−1.46	9.59

A one-way ANOVA was also performed to examine physical exercise involvement total scores among different gender roles within masculine sports. The results showed significant differences in physical exercise involvement among undergraduates with different gender roles: *F*_(3, 227)_ = 16.796, *p* < 0.001, η^2^ = 0.405, indicating a strong effect size. *Post-hoc* multiple comparisons revealed that undergraduates with an androgynous gender role (*M* = 45.17, *SD* = 15.699) scored significantly higher than those with a feminine role (*M* = 29.55, *SD* = 12.137, *p* < 0.001) and an undifferentiated role (*M* = 22.83, *SD* = 8.095, *p* < 0.001). Additionally, undergraduates with a masculine role (*M* = 44.80, *SD* = 3.114) scored significantly higher than those with a feminine (*p* = 0.015) and undifferentiated role (*p* < 0.001). No significant difference was observed between individuals with feminine and undifferentiated gender roles (*p* = 0.075) (see Tables 10, 11).

**Table 10:** Total exercise involvement score differences in masculine sports by gender role.

**Project type**	***M* ±*SD***	***df***	***F***	***p***	**η^2^**
Androgynous	45.17 ± 15.699	3	16.796	0.000	0.405
Feminine	29.55 ± 12.137				
Masculine	44.80 ± 3.114				
Undifferentiated	22.83 ± 8.095				

**Table 11:** *Post-hoc* multiple comparisons of total exercise involvement score across gender roles.

**Item**	**Mean difference**	***SE***	***p***	**95%** ***CI***	
				**Lower**	**Upper**
Androgynous—Feminine	15.67	3.580	0.000	8.54	22.80
Androgynous—Masculine	0.31	5.964	0.959	−11.57	12.19
Androgynous—Undifferentiated	22.41	3.399	0.000	15.64	29.19
Feminine—Masculine	−15.36	6.158	0.015	−27.63	−3.09
Feminine—Undifferentiated	6.74	3.729	0.075	−0.69	14.17
Masculine—Undifferentiated	22.11	6.055	0.000	10.04	34.17

Regarding persistence, the ANOVA results showed no significant differences among undergraduates with different gender roles: *F*_(3, 227)_ = 2.385, *p* = 0.076, η^2^ = 0.088 (see Table 12).

**Table 12:** Persistence differences in masculine sports by gender role.

**Project type**	***M* ±*SD***	**d*f***	***F***	***p***	**η^2^**
Androgynous	11.69 ± 6.985	3	2.385	0.076	0.088
Feminine	8.10 ± 4.494				
Masculine	12.60 ± 1.949				
Undifferentiated	8.83 ± 4.806				

For concentration, the ANOVA results indicated significant differences among gender roles: *F*_(3, 227)_ = 4.127, *p* = 0.009, η^2^ = 0.143. *Post-hoc* multiple comparisons revealed that undergraduates with androgynous (*M* = 11.17, *SD* = 6.985, *p* = 0.004), feminine (*M* = 11.65, *SD* = 4.902, *p* = 0.004), and masculine gender roles (*M* = 12.20, *SD* = 2.588, *p* = 0.046) scored significantly higher than those with undifferentiated gender roles (*M* = 6.88, *SD* = 3.379). However, no significant differences were observed between undergraduates with androgynous and feminine roles (*p* = 0.777), androgynous and masculine roles (*p* = 0.700), or feminine and masculine roles (*p* = 0.834) (see Tables 13, 14).

**Table 13:** Concentration in masculine sports by gender role.

**Project type**	***M* ±*SD***	**d*f***	***F***	***p***	**η^2^**
Androgynous	11.17 ± 6.985	3	4.127	0.009	0.143
Feminine	11.65 ± 4.902				
Masculine	12.20 ± 2.588				
Undifferentiated	6.88 ± 3.379				

**Table 14:** *Post-hoc* multiple comparisons of concentration across gender roles.

**Item**	**Mean difference**	***SE***	***p***	**95%** ***CI***	
				**Lower**	**Upper**
Androgynous—Feminine	−0.44	1.554	0.777	−3.54	2.65
Androgynous—Masculine	−1.00	2.588	0.700	−6.16	4.16
Androgynous—Undifferentiated	4.32^*^	1.475	0.004	1.38	7.26
Feminine—Masculine	−0.56	2.673	0.834	−5.89	4.76
Feminine—Undifferentiated	4.76^*^	1.618	0.004	1.54	7.99
Masculine—Undifferentiated	5.32^*^	2.628	0.046	0.09	10.56

*p* < 0.05 statistically significant and marked with an asterisk (*).

Regarding value cognition, the ANOVA results revealed significant differences among undergraduates with different gender roles: *F*_(3, 227)_ = 39.245, *p* < 0.001, η^2^ = 0.614, indicating a strong effect size. *Post-hoc* multiple comparisons showed that undergraduates with androgynous gender roles (*M* = 13.97, *SD* = 4.975) scored significantly higher than those with feminine (*M* = 4.55, *SD* = 3.103, *p* < 0.001) and undifferentiated roles (*M* = 4.13, *SD* = 2.833, *p* < 0.001). Similarly, undergraduates with masculine roles (*M* = 13.00, *SD* = 2.449) scored significantly higher than those with feminine (*p* < 0.001) and undifferentiated roles (*p* < 0.001). No significant difference was observed between feminine and undifferentiated roles (*p* = 0.720) or between masculine and androgynous roles (*p* = 0.606) (see Tables 15, 16).

**Table 15:** Value cognition in masculine sports by gender role.

**Project type**	***M* ±*SD***	**d*f***	***F***	***p***	**η^2^**
Androgynous	13.97 ± 4.975	3	39.245	0.000	0.614
Feminine	4.55 ± 3.103				
Masculine	13.00 ± 2.449				
Undifferentiated	4.13 ± 2.833				

**Table 16:** *Post-hoc* multiple comparisons of value cognition across gender roles.

**Item**	**Mean difference**	***SE***	***p***	**95%** ***CI***	
				**Lower**	**Upper**
Androgynous—Feminine	9.38^*^	1.112	0.000	7.16	11.60
Androgynous—Masculine	0.96	1.853	0.606	−2.73	4.65
Androgynous—Undifferentiated	9.80^*^	1.056	0.000	7.69	11.90
Feminine—Masculine	−8.42^*^	1.914	0.000	−12.23	−4.61
Feminine—Undifferentiated	0.42	1.159	0.720	−1.89	2.73
Masculine—Undifferentiated	−8.84^*^	1.881	0.000	−12.58	−5.09

*p* < 0.05 statistically significant and marked with an asterisk (*).

For participation autonomy, the ANOVA results showed significant differences among gender roles: *F*_(3, 227)_ = 10.965, *p* < 0.001, η^2^ = 0.308, reflecting a strong effect size. *Post-hoc* multiple comparisons revealed that individuals with androgynous gender roles (*M* = 8.34, *SD* = 3.949) scored significantly higher than those with feminine (*M* = 5.25, S*D* = 3.905, *p* = 0.003) and undifferentiated roles (*M* = 3.00, *SD* = 2.485, *p* < 0.001). Similarly, those with masculine roles (*M* = 7.00, *SD* = 3.391) scored significantly higher than participants with undifferentiated roles (*p* = 0.018), while those with feminine roles (*p* < 0.001) scored significantly higher than undifferentiated roles (*p* = 0.034). No significant difference was observed between feminine and masculine roles (*p* = 0.288) or between masculine and androgynous roles (*p* = 0.456) (see Tables 17, 18).

**Table 17:** Participation autonomy in masculine sports by gender role.

**Project type**	***M* ±*SD***	**d*f***	***F***	***p***	**η^2^**
Androgynous	8.34 ± 3.949	3	10.965	0.000	0.308
Feminine	5.25 ± 3.905				
Masculine	7.00 ± 3.391				
Undifferentiated	3.00 ± 2.485				

**Table 18:** *Post-hoc* multiple comparisons of participation autonomy across gender roles.

**Item**	**Mean difference**	***SE***	***p***	**95%** ***CI***	
				**Lower**	**Upper**
Androgynous—Feminine	3.14^*^	1.017	0.003	1.12	5.17
Androgynous—Masculine	1.27	1.694	0.456	−2.11	4.64
Androgynous—Undifferentiated	5.43^*^	0.965	0.000	3.50	7.35
Feminine—Masculine	−1.87	1.749	0.288	−5.36	1.61
Feminine—Undifferentiated	2.29^*^	1.059	0.034	0.18	4.40
Masculine—Undifferentiated	4.16^*^	1.719	0.018	0.73	7.58

*p* < 0.05 statistically significant and marked with an asterisk (*).

## 4 Discussion

The present study categorized the sports as masculine sports, feminine sports, and gender-neutral sports while undergraduate gender roles as androgynous, feminine, masculine, and undifferentiated. And then analyze the associations between gender roles and PA-related characters, including project types and physical exercise involvement propose targeted teaching strategies to improve female undergraduates' participation in physical activities.

The comparison of PA levels and physical exercise involvement revealed significantly lower engagement among female undergraduates compared to their male counterparts, as indicated by the scores on the PARS-3 and PEIS. Female participants expressed particular dissatisfaction with dimensions related to persistence, concentration, value cognition, and participation autonomy. Data in detail showed that female undergraduates performed lower enthusiasm, initiative, and energy in classes than their male peers. Along with lower satisfaction and perceived exercise effectiveness, female undergraduates fell into a negative feedback loop of PA participation, where negative outcomes lead to subsequent disengagement and perpetuate the cycle. These findings align with existing evidence suggesting that male undergraduates tend to engage in physical activity more actively and autonomously than their female peers (Yu et al., 2024). However, no significant sexual differences were observed in sports preferences, as the distribution of choices for masculine, feminine, and gender-neutral sports showed no statistical significance based on chi-square tests. Traditionally, masculine sports involve intense physical confrontation (Xiong, 2013), leading to higher PA levels. The “male-oriented” characteristics of Clément-Guillotin et al. (2012) agitate less participation among females. On the contrary, feminine sports are often static (Hardin and Greer, 2009), requiring less physical confrontation which wins great favor from females for providing more options for time and PA intensity. The present results could account for the changes in sports-related social stereotypes and the guidance of teachers on sports choosing (Vaquero-Cristóbal et al., 2024). As social progress, females are encouraged to participate in social competition (Cassar and Rigdon, 2021) and be free to express aggression and ambiguity (Liu et al., 2011). In addition, PE teachers gain awareness to encourage females taking more exercise and feel free to take part in masculine sports for better PA volume and physical fitness results (Leaper and Brown, 2014).

The disadvantage of female undergraduates' PA levels accounts for the gender-role variance given no differences were observed in sports type choice and both male and female undergraduates share equal opportunities for PE courses on campus. The present data analyzed with sex showed that males were more likely to identify as masculine or androgynous, and females exhibited a higher frequency of feminine and undifferentiated gender roles compared to males. Significant differences in PA levels were observed among different gender roles. In line with previous studies about gender roles in sports, we also observed that individuals with androgynous roles exhibited the highest PA levels, followed by those with masculine roles, while those with feminine and undifferentiated roles had lower levels. Prior studies suggest that individuals with androgynous traits tend to engage in a wider range of exercises, maintain more structured exercise habits, and develop better interpersonal relationships (Xu et al., 2010). Those with masculine traits exhibit strong competitiveness and persistence in exercise (Yao et al., 2008), while individuals with feminine traits, although capable of forming stable exercise relationships, are influenced by traditional gender norms to exhibit lower frequency and intensity in exercise behavior (Wang and Zhang, 2012). Individuals with undifferentiated traits are regarded as having the least desirable personality type, often associated with poor self-regulation, significant social barriers, lower subjective wellbeing, and weak exercise persistence (Xu et al., 2010). They are in line with support that advocates reducing gender stereotypes and role constraints on females in educational contexts.

What's more, the present results showed that female undergraduates exhibited the lowest overall PA levels in masculine sports, and within this group, those with androgynous gender roles performed best both in cognition and behavior while those with feminine roles showed notably lower scores in the value cognition aspect of physical exercise involvement. Female undergraduates with androgynous roles demonstrated higher physical exercise involvement than other female groups because of a better understanding of the value, significance, and functions of sports. They generally gained a positive and enjoyable PA attitude and exercised intrinsic motivation, such as health pursuit. The unsatisfied performance of female with feminine roles in masculine sports may account for the conflicts between their gender identity and the gendered nature of the sports. When participating in masculine sports, such as football, basketball, and combat sports, female undergraduates often seek value or recognition “like males” (Hardin and Greer, 2009; Zhan, 2016). This perspective of being “subordinates” or “imitators” directly leads to gender role conflicts (McGannon and Busanich, 2010), causing instability in their physical exercise involvement and limited performance in achieving the high-intensity PA required in masculine sports (Moylan et al., 2024). Therefore, gender role identity and gender categorization affect the PA levels of female undergraduates and hinder the long-term development of their motor skills, reinforcing the gendered characteristics of sports and undermining the inclusivity and equality of these activities.

Back to the implementation of PE in the universities, gender compatibility in sports could be increased. It is also important to enhance female undergraduates satisfaction, value recognition, and sense of agency in masculine and gender-neutral sports while reducing gender role conflict the following anxiety. It is also viable to incorporate male-oriented elements into feminine sports to increase the intensity of physical activities, thereby improving the overall PA levels of female undergraduates.

## 5 Research limitations

Owing to the small sampling range, this study was limited to 439 undergraduates from universities in Shanghai, which restricted the generalizability of the findings. Future studies could expand the sample size to improve representativeness. PA levels in this study were measured using self-reported questionnaires, increasing the risk of estimation bias. Future research could employ wearable devices such as accelerometers or heart rate monitors for more precise analyses. The bidirectional relationship between androgyny and sports participation requires further investigation by scholars.

## 6 Conclusion

Female undergraduates gained significantly lower PA levels and physical exercise involvement compared to those of their male peers. The stereotypes of gender and the perceived characteristics of sports interacted with female undergraduates' PA level and physical exercise involvement which provide a potential approach to reverse the disadvantage of female undergraduates in PA.

## Data Availability

The raw data supporting the conclusions of this article will be made available by the authors, without undue reservation.
