# Correction: Teaching strategies for promoting female college students' physical activity

**DOI:** 10.3389/fpsyg.2025.1663108

**Published:** 2025-08-05

**Authors:** Ji Ma, Yuqiang Guo, Chao Zhu, Chunyuan Wen, Qiaoqiao Deng, Xiao Ma

**Affiliations:** School of Physical Education, Shanghai University of Sport, Shanghai, China

**Keywords:** gender roles, exercise involvement, sports projects, physical activity, female undergraduates

The funder “Shanghai Higher Education Institutions Young Faculty Development and Funding Program for the Year 2024” to “Xiao Ma” was erroneously omitted.

The original version of this article has been updated.

